# Development, structure and evolutionary significance of seed appendages in *Salix matsudana* (Salicaceae)

**DOI:** 10.1371/journal.pone.0203061

**Published:** 2018-09-04

**Authors:** Jianxia Li, Xiaofei Xia, Shenjian Xu, Jiayue Wu, Linlin Peng, Liangcheng Zhao

**Affiliations:** 1 Lab of Systematic Evolution and Biogeography of Woody Plants, College of Nature Conservation, Beijing Forestry University, Beijing, China; 2 Beijing Museum of Nature History, Beijing, China; USDA-ARS, UNITED STATES

## Abstract

The seeds of *Salix* and *Populus* (Salicaceae) are characterized by having numerous long hairs which loosely accompanying the seeds and a small annular appendage which surrounding the base of the seed along with tufted hairs. In this study, the complete development and detailed structure of the hairs and annular appendage in *Salix matsudana* were investigated using standard techniques for plant anatomy and histochemistry. The results show that the hairs originate successively from the single epidermal cells of the placenta (in megaspore mother cell phase) and funiculus (in eight-nucleate phase), and that their development consists of a progressive increase in cell size and an absence of cell division. The annular appendage is initiated from four to five rows of cells at the distal end of the funiculus in octant proembryo phase and its development is characterized by reactivated meristematic activity and a size increase of these cells. The initiation and development of the hairs are irrelevant to ovule development but fertilization and a developed embryo is necessary for the annular appendage to occur. Considering the reliable fossils, we inferred that the feature of seeds surrounded by long hairs is an ancestral character, and that the detachment of hairs from the funiculus and the occurrence of an annular appendage with tufts of hairs may be the more derived states for seed dispersal in *Salix* and *Populus*.

## Introduction

The woody family Salicaceae traditionally constitutes two genera, *Salix* and *Populus*, both occurring predominantly in temperate and cold regions of the Northern Hemisphere, but it was recently enlarged to include a large number of tropical genera formerly placed in the family Flacourtiaceae [1, 2]. The largest genus *Salix*, commonly known as willows, consists of about 450–520 species and is distributed mainly in the Northern Hemisphere, from arctic through temperate latitudes, and a few are found in South America and only one polymorphic species naturally grew in Africa [3–5]. Because of their fast growth rate, high habitat adaptability, and versatile uses (e.g. ornamental, timber, medicine, bioenergy), *Salix* species are economically and ecologically one of the most important groups of trees [6, 7].

**Fig 1:**
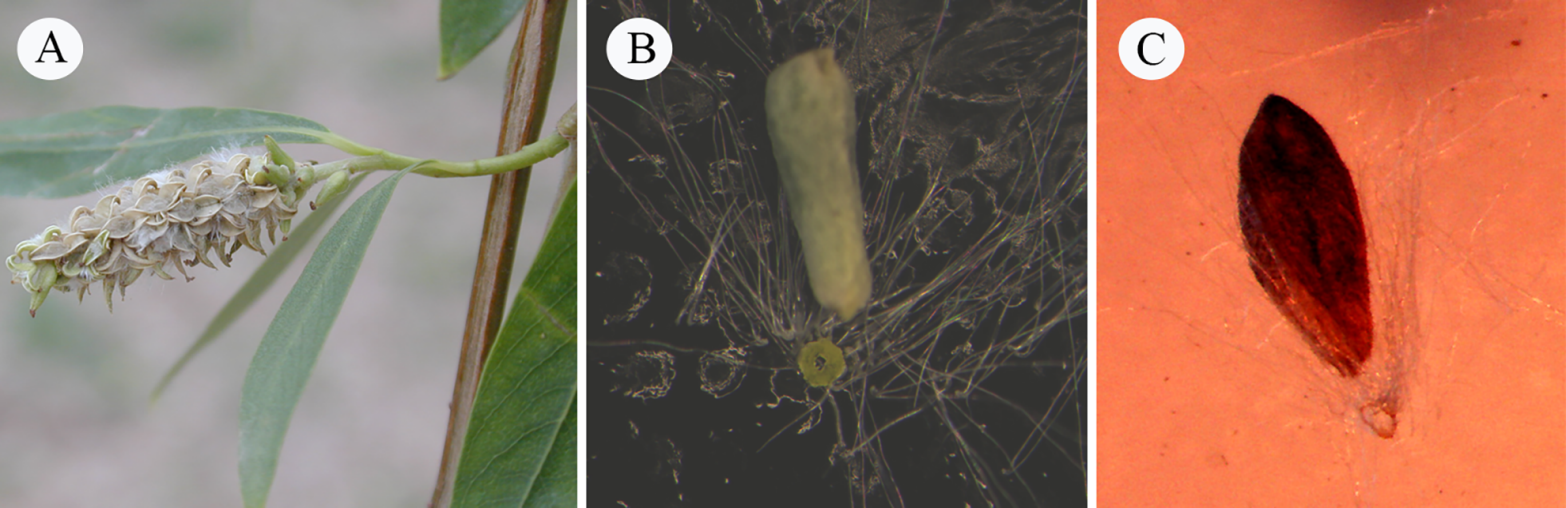
Infructescence and seed of *Salix matsudana*, and seed of *Populus lasiocarpa*. **A.** Mature infructescence of *Salix matsudana*, showing numerous whitish, silky hairs in each dehiscent capsule. **B.** Mature seed of *Salix matsudana*, showing a detached annular appendage with tufts of hairs surrounding the base of the seed. **C.** Mature seed of *Populus lasiocarpa*, showing a detached annular appendage with tufts of hairs surrounding the base of the seed.

In reproductive features, *Salix* as well as *Populus* is characterized by the dioecious and highly reduced flowers and small seeds with surrounding appendage in the form of numerous whitish, silky hairs, which exceed the length of the seed and spread out at maturity of the capsule (Fig 1A). This kind of hair has attracted attention not only for its well-known function in seed dispersal [8, 9], but for the environmental annoyance when floating in the air, which may cause direct or indirect discomfort in humans [10]. Different terms have been used for describing these hairs, such as coma, cotton, down, flocculus, pappus, plume and tufts [8, 9, 11–18]. Although the development of these hairs in the two genera have been to a lesser or greater degree involved or mentioned in several studies on floral and seed development [11–13, 16,17], few observation has been made to correlate its developmental features with those of the ovule at different stages, and a detailed study on the complete ontogeny of this hair appendage is still lacking.

In addition to the numerous hairs surrounding the seeds, in both *Salix* and *Populus*, there is another unique annular appendage at the radical end of the seed, which separates the seed from the funiculus and easily breaks away from the seed (Fig 1B and 1C). This annular appendage is very tiny and often concealed by long hairs so that it usually was overlooked by workers in the past. The morphology and development of this appendage have only been described in some detail for the African willow species, *Salix mucronata* Thunb. [17], and little is known about its complete development, detailed structure and the correlation between its development and the development of the ovule, especially in other members of the genus.

*Salix matsudana* Koidz., commonly known as Chinese willow, is a large, deciduous, rapidly growing tree native to northeastern China and is being cultivated widely as ornamental and soil stabilization trees[4]. The flowers of this species are borne in catkins produced early in spring (middle of March) and its fruits mature in late April. Here we use standard techniques for plant anatomy and histochemistry to investigate the whole ontogeny and detailed structures of the two seed appendages in *S*. *mutsudana*, and intend to show how they are initiated and how they complete their development, especially to establish the association between their development and the development of the ovules. The results can provide not only new information on the developmental and structural features of the two appendages, but also new insights into their evolution in Salicaceae. In addition, different processes in their development will be characterized based on distinguishing morphological and anatomical features that can be used in future developmental genetic studies.

## Materials and methods

The tree of *Salix mutsudana* sampled is located on the campus of Beijing Forestry University, Beijing, China. The location is not privately owned or protected and we were allowed by the administrator to collect the required samples of plant material. To study the complete ontogeny, the female flowers and fruits were collected at various developmental stages and the collections began from the middle of March, and were made at intervals of 3–5 days until the end of April. For comparing the development of unfertilized ovules, some female catkins were labeled and bagged in pre-anthesis stage so that the ovules were not available for pollination. For comparing some seed structures of *Salix* with those in *Populus*, mature fruiting material of *Populus* × *tomentosa* Carrière was obtained from the Museum of Beijing Forestry University, Beijing China, and mature seeds of *P*. *lasiocarpa* Oliv. were provided by Shangce, a researcher of *Populus* at Beijing Forestry University.

The collected samples of flowers and young fruits were fixed in FAA (formalin-acetic acid-50% alcohol, 1:1:18). For paraffin wax sectioning, fixed samples were dehydrated in an ethanol series (50, 70, 85, 95, and 100%) for 2 h in each bath. The dehydrated samples were cleared with ethanol-xylol (1:1) for 2 h, and placed in absolute xylene for 2 h. The samples were then transferred to a mixture of xylene and wax dust at 38°C overnight, and embedded in paraffin wax (12 h at 59°C; three paraffin changes). Sections of 8 μm thick were obtained with a Leica RM2126 rotary microtome. The sections then were stained with toluidine blue O (TBO) [19] for structural characterization and periodic acid-Schiff 's reagent (PAS) for storage polysaccharides (starches) and structural polysaccharides (cellulose of the cell wall) [20]. At the same time, fresh samples were cut using a cryomicrotome (CM1850, Leica Microsystems) to detect cutin with Sudan III and lignin using phloroglucinol. The sections of fixed material were mounted on slides using synthetic resin and those of fresh material in glycerinated gelatin. All sections were observed under the light microscope (LM) (Olympus BX-51), and photographed using an Olympus DP72 photomicrography system.

For scanning electron microscope (SEM) observations, the fixed material was dehydrated in an ethanol series, critical point dried, attached to stubs with double-sided sticky tape, coated with gold, and examined using a SEM (HITACHI S-3400) at 20–30 kV at Beijing Museum of Nature History.

All figures were prepared with Adobe Photoshop v. 7.0 software.

## Results

### Hair appendage development

In order to investigate the correlation between the development of the hair appendage and the development of the ovule in *S*. *matsudana*, the whole developmental process was divided into two stages: the pre-fertilization stage and the post-pollination stage.

#### Pre-fertilization stage

**Fig 2:**
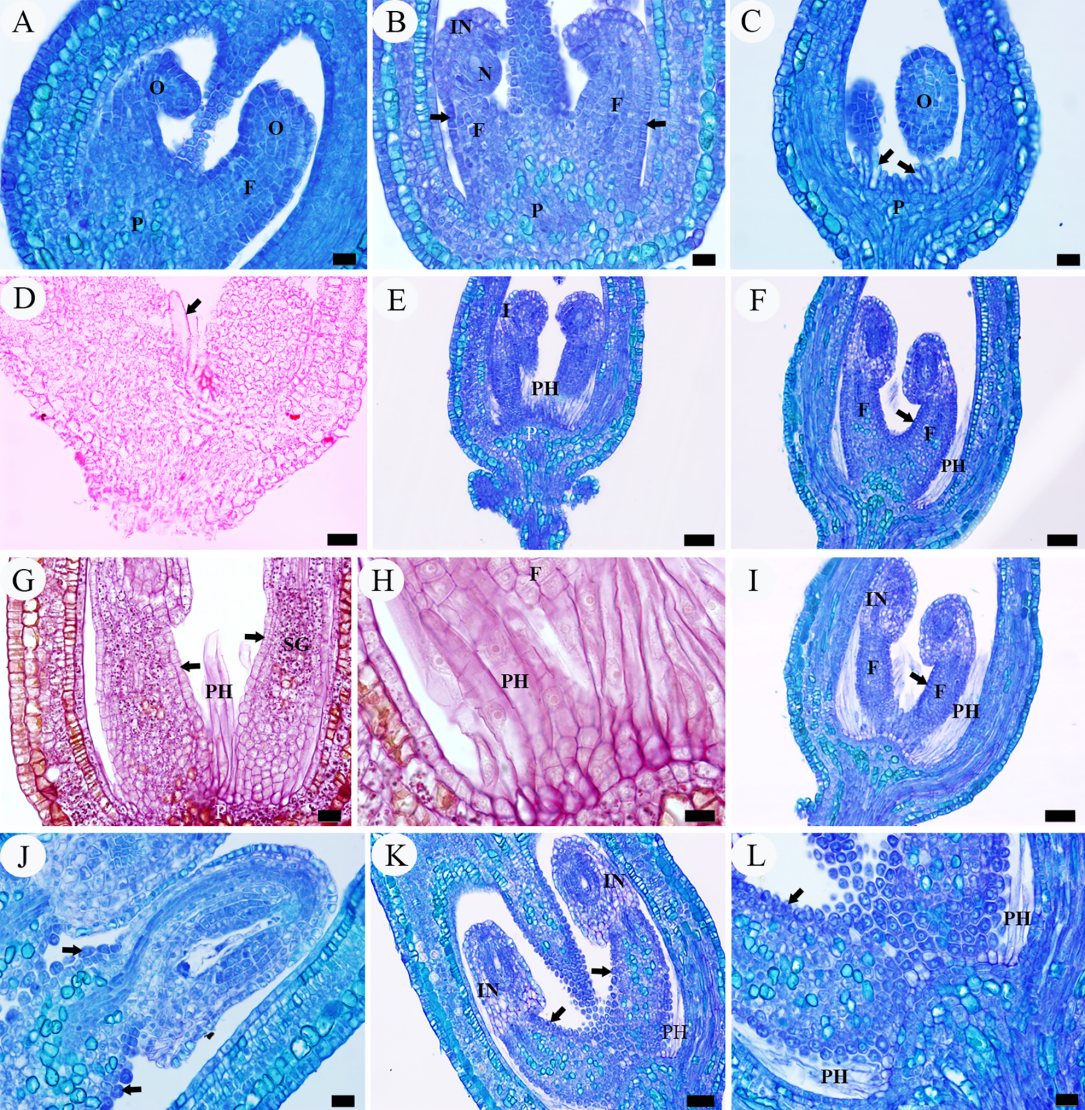
LM micrographs of intra-ovarian hair development in pre-fertilization stage. A, archesporial cell phase, showing two young ovules with basal placenta. B–D, megaspore mother cell phase. B, young ovules, showing the well-developed funiculus with a single layer compact epidermis (arrow). C, showing finger-like protrusions (arrow) derived from the placenta epidermal cells. D, showing placenta epidermal protrusions (arrow), indicating the presence of cutin in cell wall. E–I, four-nucleate to functional megaspore phase. E, F, showing elongated placental hairs. G, showing funicular cortical cells with starch grains accumulated but epidermal cells (arrow) without starch grains. H, showing ribbon-like placental hairs. I, showing long placental hairs and funicular epidermis (arrow). J–L, eight-nucleate phase, showing the enlarged and loosely arranged epidermal cells of the funiculus (arrow). Scale bars: A–C, G, J, L = 20 μm; D–F, I, K = 50 μm; H = 10 μm. *Abbreviations*. F, funiculus; FC, funiculus cortical cell; IN, integument; N, nucellus; O, ovule; P, placenta; PH, placental hair; SG, starch granule. All are longitudinal sections. A–C, E, F, I–L stained with TBO, D stained with Sudan III, and G, H stained with PAS.

**Fig 3:**
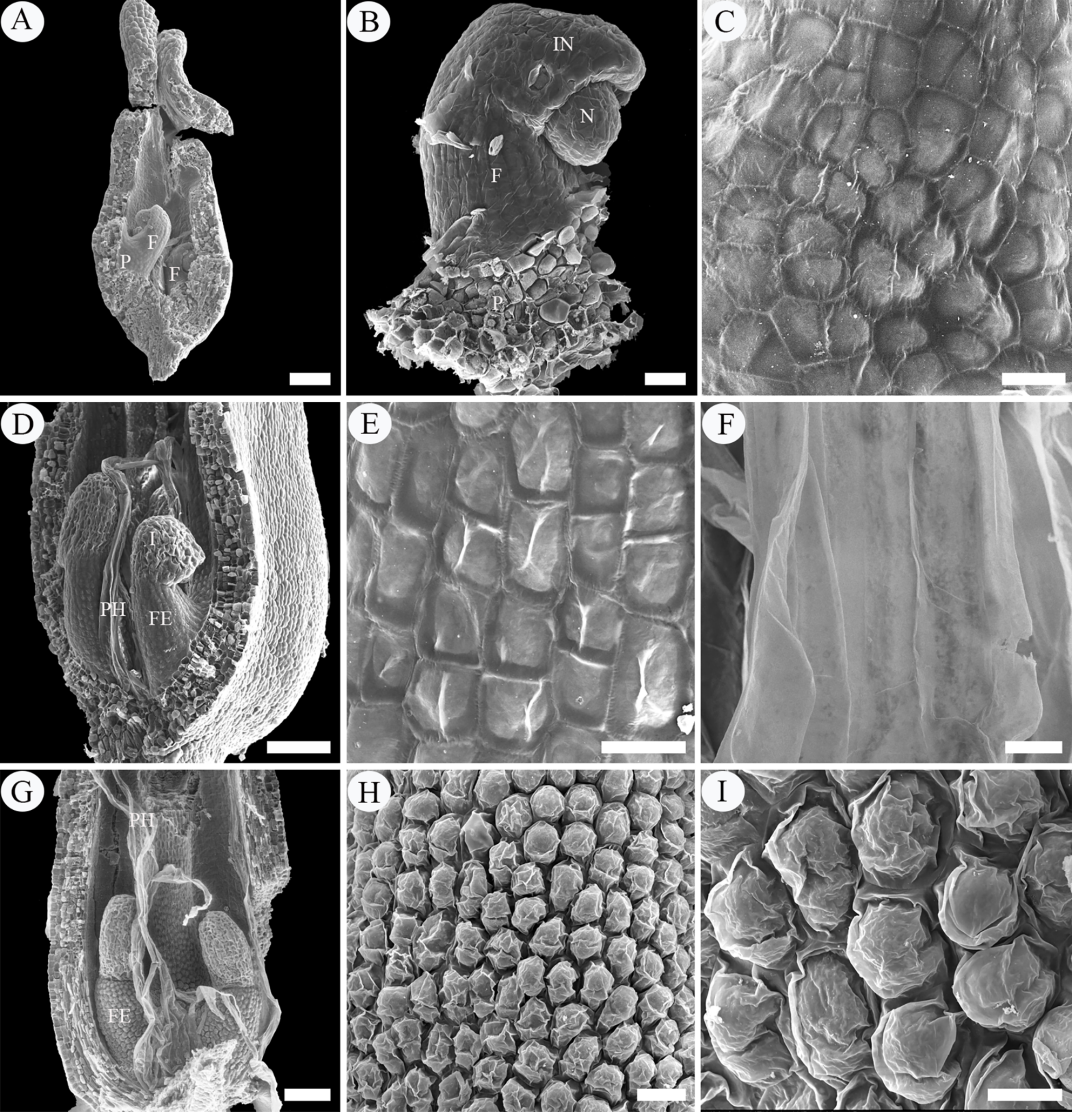
SEM micrographs of intra-ovarian hair development in pre-fertilization stage. A–C, archesporial cell to megaspore mother cell phase. A, two young ovules with basal placenta. B, an ovule, showing the well-developed funiculus. C, enlargement of B, showing the funicular epidermal cells. D, E, four-nucleate to functional megaspore phase. D, showing elongated placental hairs and well-developed funiculus with epidermal cells. E, enlargement of D, showing the rectangular and tightly arranged funicular epidermal cells. F–I, eight-nucleate phase. F, ribbon-like placental hairs. G, showing long placental hairs and swelled funicular epidermis. H, I, enlargement of G, showing the enlarged and loosely arranged epidermal cells of funiculus. Scale bars: A, D, G = 100 μm; B, H = 20 μm; C, E, F, G, I = 10 μm. *Abbreviations*. F, funiculus; FE, funiculus epidermis; IN, integument; N, nucellus; O, ovule; P, placenta; PH, placental hair.

A complete ovary of *Salix matsudana* comprised of a single locule with two ovules (rarely three) with basal placentation (Figs 2A and 3A). During the phases of archesporial cell and megaspore mother cell of the megagametophyte, the young ovules are approximately 0.12 mm (excluding the funiculus) in length, consisting of a well developed funiculus, two to four cell-layered developing integument and a nucellus about one-half of the ovule length (Figs 2B and 3B). At this stage, these ovules are hemitropous due to the bending of the funiculus, with the micropyle oriented toward the placenta on the side of the funiculus toward the near ovary wall. The funiculus is stout, cylindrical, about 60–70 μm in diameter, composed of five to seven cell-layered parenchymatous tissue (except being thicker at the proximal end), with a single layer compact epidermis (Figs 2B and 3B). In transverse section and surface view the epidermal cells of the funiculus are polygonal or short rectangular with dense cytoplasm, easily distinguishable from the funicular cortical parenchymatous cells and the outermost layer cells of the integument, which having larger vacuoles and less cytoplasm (Figs 2B, 3B and 3C). In the megaspore mother cell phase or even earlier, the placenta epidermal cells around the funicular proximal end initially protruded above the placenta surface and began to elongate toward the ovule, which are about 18–38 μm long and 5–7 μm wide, appearing as finger-like protrusions perpendicularly to the ovary wall (Fig 2C). Each protrusion resulted from the elongation of individual epidermal cells, with the nucleus located basally to centrally. These cells are more vacuolated and more lightly stained than other epidermal cells of the ovary (Fig 2C). With Sudan III, the periphery of the protrusion is dark red, indicating the presence of lipophilic material such as cutin in its cell wall (Fig 2D). The duration of the two phases was about six days (March 16–March 22) (Fig 7).

During the four-nucleate (tetrad) and functional megaspore phases of the megagametophyte, the protrusions continued to enlarge in size, up to about 280 μm in length and 10 μm in width, developing into flattened ribbon-like hairs (placental hairs). They are thin-walled, mononuclear, much vacuolated with little cytoplasm (Figs 2E–2H, 3D and 3F). These hairs grew very rapidly so that some of them have exceeded the length of the developing ovule by this time (Fig 3D). At this stage, the ovules attained a complete anatropous state. The funiculus increased slightly in diameter and its epidermal cells are still arranged relatively tightly in the form of a rectangular or square outline, with conspicuous nucleus and dense cytoplasm (Fig 2E, 2F and 2I). Stained with PAS, a noteworthy feature is that in the cortical cells of the funiculus and placenta, a substantial amount of starch grains accumulated but there are no starch grains in both the funiculus epidermal cells and the placental hairs (Fig 2G and 2H). Compared with the funiculus, the integument epidermal cells showed an obvious vacuolization of their cytoplasm with a few starch grains (Fig 2E–2G and 2I). The duration of the two phases was about six days (March 23–March 28) (Fig 7).

During the eight-nucleate phase (Fig 2J), the placental hair cells continued to grow toward the ovule rapidly (Fig 2K and 2L), up to about 760 μm long and 13 μm wide. By now they have much exceeded the developing ovule in length and completely inclosed it (Fig 3G). It is important to note that in this phase the funiculus has undergone a remarkably thickness increase up to 90–120 μm in diameter and the epidermal cells on all sides of the funiculus began to enlarge outwards, characterized by their shape changing from rectangular to round or irregular and their arrangement from tight to very loose with large intercellular spaces, appearing as numerous separate papillae, forming hair primordium (Figs 2J–2L and 3G–3I). These primordium cells have denser cytoplasm, distinguishable from the inner cortical cells (Fig 2J–2L). Their distinctive shape and arrangement are also obviously distinguished from the epidermal cells of the integument, which are larger, much vacuolated and tightly arranged, often showing a wrinkle outline (Figs 2K and 3G). The duration of this phase was about five days (March 29–April 2) (Fig 7).

#### Post-fertilization stage

**Fig 4:**
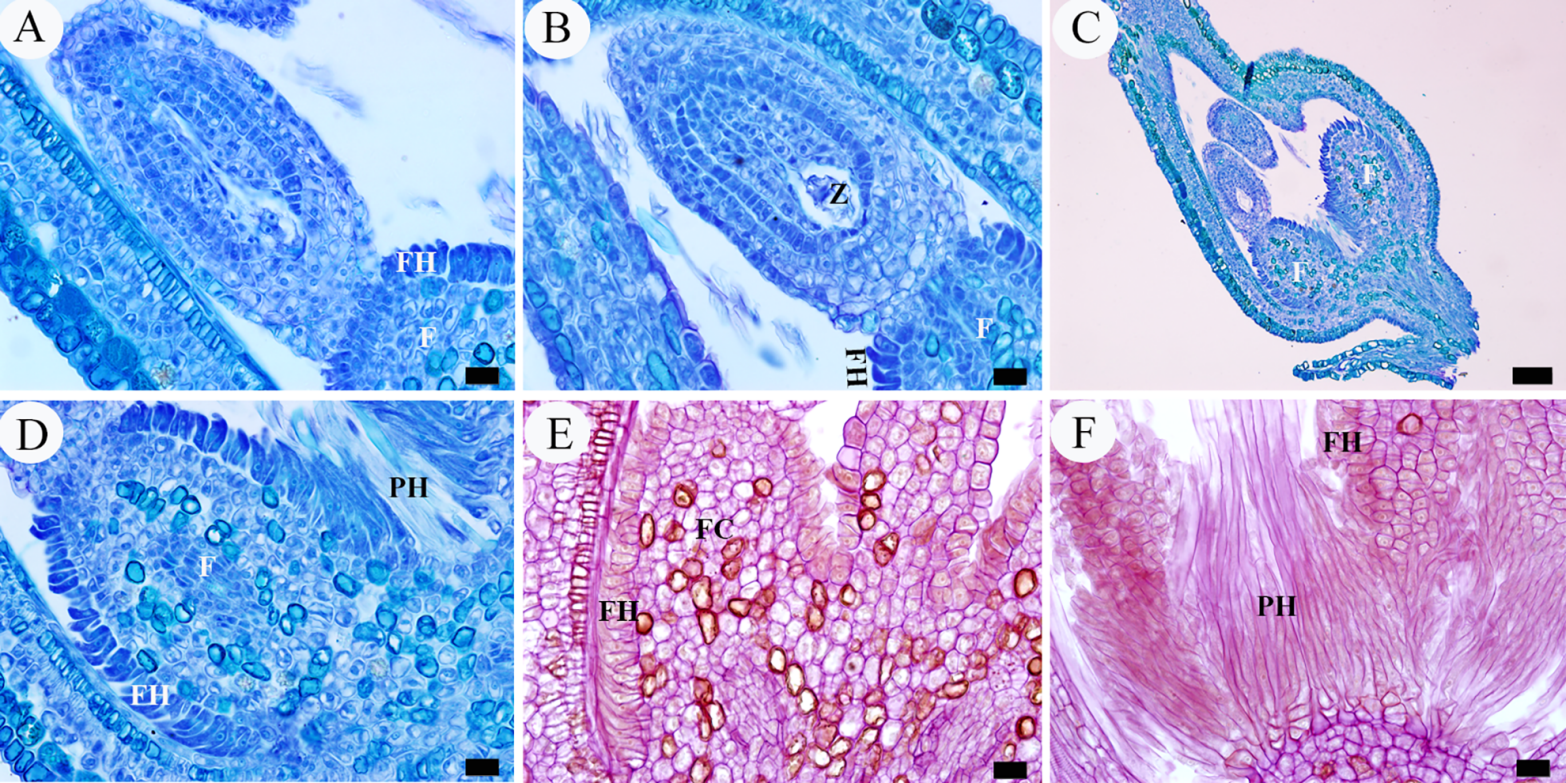
LM micrographs of intra-ovarian hair development in fertilization stage. A–D, showing the sickle-shaped or crescentic funicular hair cells. E, showing the funicular cortical cells with starch grains remarkably decreased. F, showing the long placental hairs without starch grains. Scale bars: A, B, D–F = 20 μm; D = 100 μm. *Abbreviations*. F, funiculus; FH, funicular hair; FC, funicular cortical cell; PH, placental hair; Z, zygote. All are longitudinal sections. A–D stained with TBO, D, F stained with PAS.

After pollination, ovule fertilization was verified by the presence of the zygote or developed endosperm (Fig 4B). At this stage, the enlarged epidermal cells (hair primordia) of the funiculus have grown obliquely upward, giving rise to curved hairs about 25–42 μm long and 5–7 μm wide (funicular hairs) (Figs 4A–4F and 6A–6C). Each funicular epidermal cell gives rise to a hair and the growth rate of the hairs in the lower (proximal) part of the funiculus is apparently faster than that in the upper (distal) part (Figs 4D and 6B). In longitudinal section, the funicular hair cells are sickle-shaped or crescentic in outline, each with a large amount of cytoplasm, many scattered small vacuoles, and a conspicuous nucleus situated at the basal end to central part of the hair (Fig 4D and 4E). At the fertilization stage, the starch grains in cortical cells of the funiculus remarkably decreased but there are still no starch grains in both the funicular and placental hairs (Fig 4E and 4F). As the elongation progressed, the funicular hair cells then have a fingerlike outline (Fig 6D and 6E).

**Fig 5:**
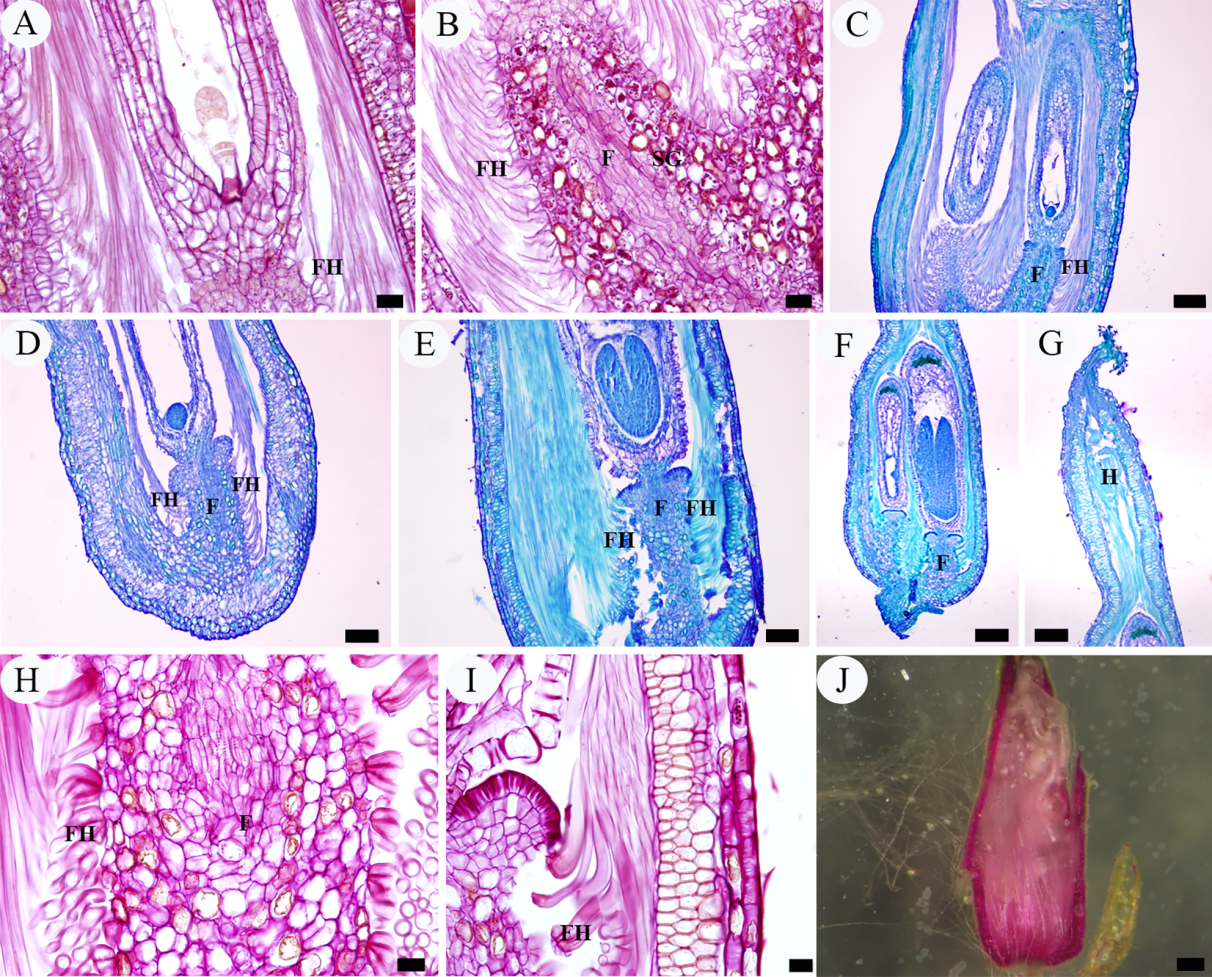
LM micrographs of intra-ovarian hair development in post-fertilization stage. A, octant proembryo phase, showing the fast-growing funicular hairs. B, enlargement of A, showing the funicular cortical cells with dense starch grains accumulated. C–D, globular embryo phase, showing funicular hairs surrounding the ovule with an U-shaped outline. E, heart-shaped embryo phase. F-I, cotyledonary embryo phase. F, G, the lower and upper part of an ovary, showing the long hairs filling the empty portions of the locule. H, showing cylindrical funicular hairs and funicular cortical cells with starch grains considerably decreased. I, near mature funicular hairs, showing detaching from funiculus. J, numerous mature hairs in fruit locule, indicating the presence of lignin in their walls. Scale bars: A, B, H, I = 20 μm; C–E = 100 μm; F, G, J = 200 μm. *Abbreviations*. FH, funicular hair; SG, starch grain. All are longitudinal sections. A, B, H, I stained with PAS, C–G stained with TBO, J stained with phloroglucinol.

In the subsequent phases of octant proembryo and globular embryo, the funicular hairs underwent very rapid elongation to 850 μm in length, now exceeding the length of the developing ovule, with their base perpendicular to the funiculus but other portion curving upward, nearly parallel to the funiculus, resulting in an U-shaped outline surrounding the ovule (Fig 5A–5E). As the funicular epidermal cells developed into hairs, the cells of the adjacent inner layer acquired a more regular arrangement and began to increase the amount of cytoplasm, thus forming a new “epidermis” of the funiculus (Fig 5C and 5D). In parallel with the elongation of funicular hairs, the placental hairs have significantly increased their length to about 1.6 mm by this time. Both the funicular and placental hairs exhibited a similar morphology, both having a broader bulbous base, a narrower lumen and a tapered tip (Fig 5C and 5D). At this time, a large amount of starch grains were accumulated again in the funiculus cortical parenchyma around the vascular bundle but still no starch grains were found in the hairs (Fig 5B). The duration of the above phases was about 11 days (April 3–April 13) (Fig 7).

**Fig 6:**
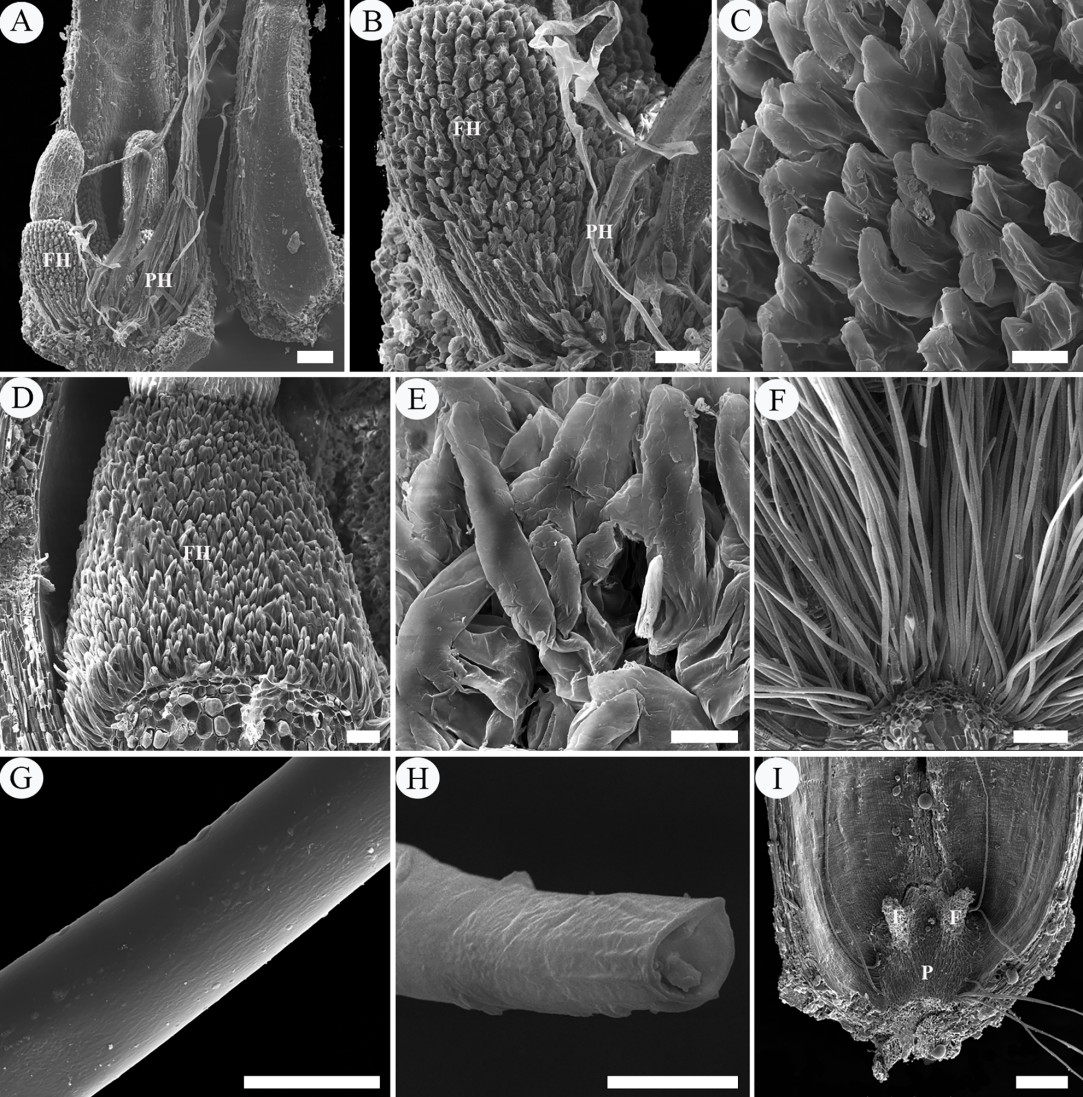
SEM micrographs of intra-ovarian hair development in post-fertilization stage. A–E, fertilization phase. A, B, showing long flattened placental hairs and elongated funicular hair cells. C, enlargement of B, showing the sickle-shaped or crescentic funicular hair cells. D, showing the finger-like funicular hair cells. E, enlargement of D. F, cotyledonary embryo phase, showing numerous cylindrical hairs. G, H, mature hairs with smooth but slightly waved surface and thicker wall. I, mature fruit, showing hairs spreading out along with mature seeds and leaving residual placenta and funiculus. Scale bars: A, F = 100 μm; B, D = 50 μm; C, E, G, H = 10 μm, I = 200 μm. *Abbreviations*. F, funiculus; FH, funicular hair; P, placenta; PH, placental hair.

**Fig 7:**
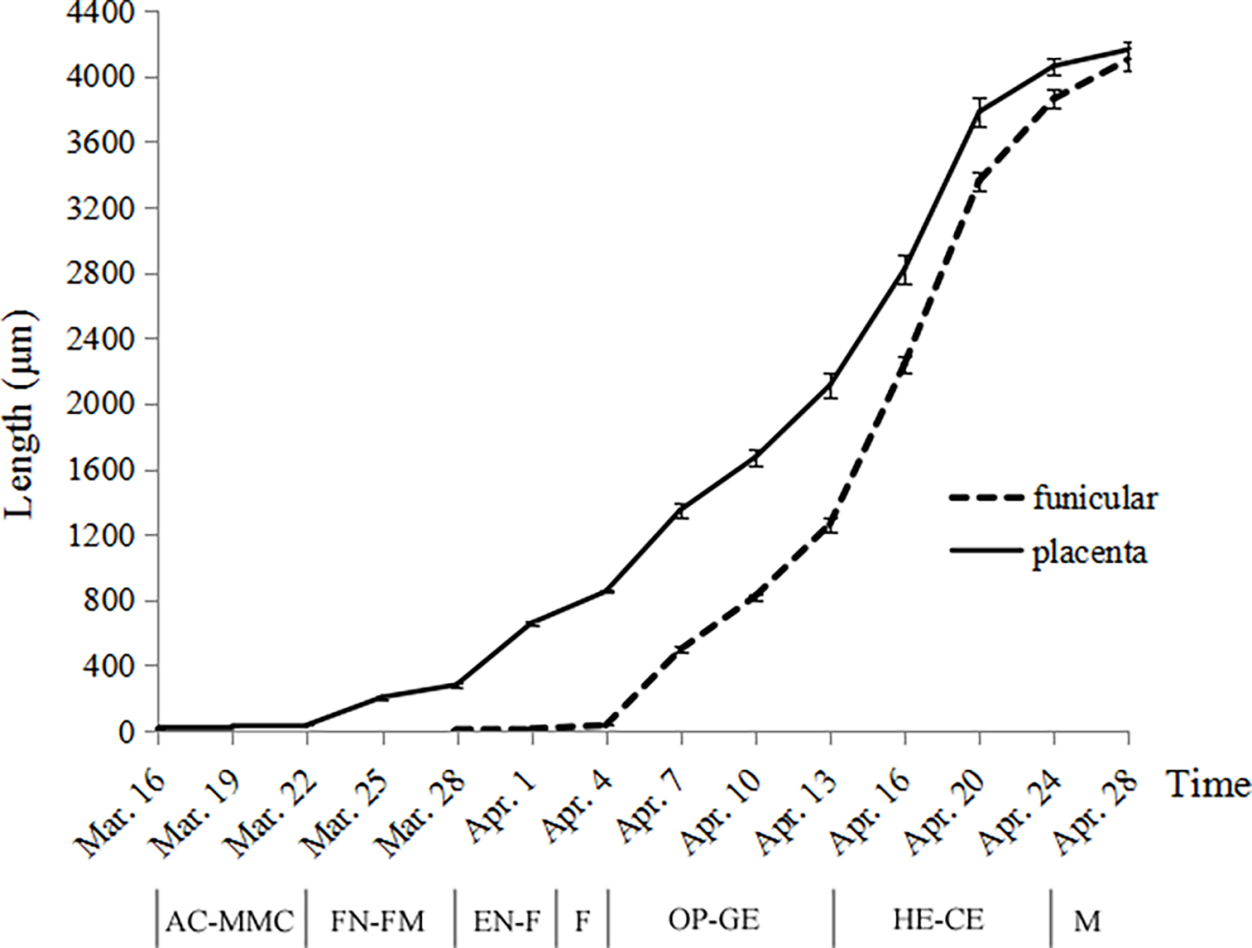
Placental and funicular hair development at different stages. Hair length (mean ± s.e.). *Abbreviations*. AC, archesporial cell; MMC, megaspore mother cell; FN, four-nucleate; FM, functional megaspore; F, fertilization; OP, octant proembryo; GE, globular embryo; HE, heart-shaped embryo; CE, cotyledonary embryo; M, mature.

The next phase of heart-shaped embryo is a continuation of the developmental period of both the placental and funicular hairs, and was characterized by their considerable increase in length and the increasing vacuolization of the cytoplasm (Fig 5E). As a result, most of the volume of the hair lumen was occupied by vacuoles. In the cotyledonary embryo phase, the fertilized ovules have nearly reached their full development, and both the funicular and placental hairs have almost reached their maximum length, filling the portions of the ovary locule not occupied by the developing seed (Fig 5F and 5G). The shape of the hairs also changed from flattened to cylindrical because the lumen became filled with air (Figs 5H, 5I and 6F). At this stage, these epidermal hairs have begun to detach from the placenta and funiculus (Fig 5I) and it is practically difficult to distinguish and separate the placental hairs and funicular hairs from each other. The starch grains in the funiculus cortical parenchyma remarkably decreased again but there are still no starch grains in both placental and funicular hairs (Fig 5H). The duration of this phase was about 11 days (April 14–April 24) (Fig 7).

During the mature seed phase, the seeds are about 1.2–1.4 mm long and have well developed cotyledons. The testa comprises only the outer tangential epidermal layer of the integument, with the outer and radial walls thickened. Both funicular and placental hairs became mature, which are about 3.8–4.2 mm in length and 5–7 μm in diameter, unbranched, aseptate, with smooth but slightly waved surface and an approximately 0.5 μm thick wall (Fig 6G and 6H). They broke away from the placenta and funiculus and almost filled the whole ovary locule. Stained with phloroglucinol, the mature hairs were red-purple, indicating the presence of lignin in their walls (Fig 5J). When the capsules dehisce and release the seeds, the placental and funicular hairs were shed along with the mature seeds, leaving the residual placenta and funiculus on the fruit wall (Fig 6I).

### Annular appendage development in fertilized ovules

**Fig 8:**
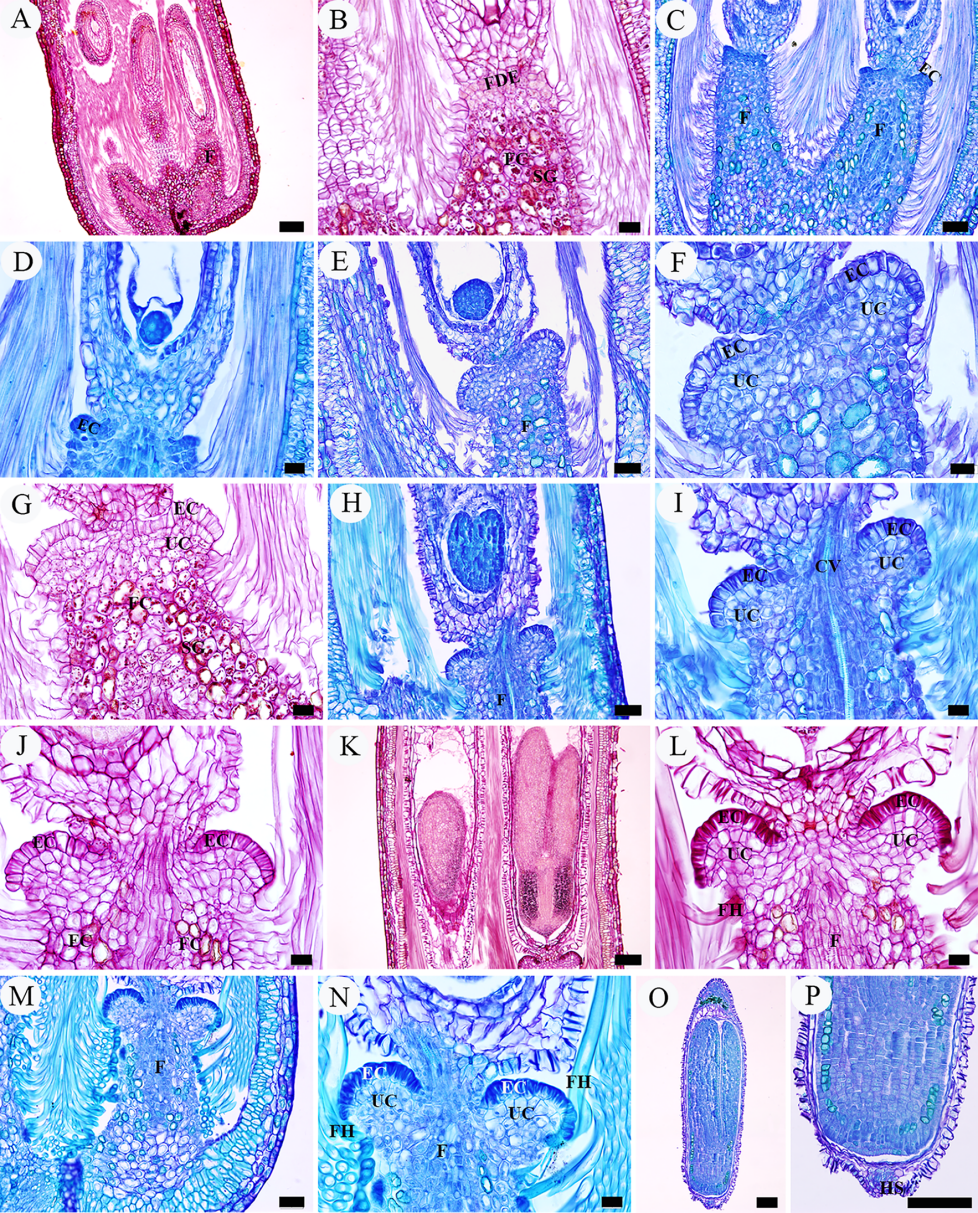
LM micrographs of annular appendage development. A, B, octant proembryo phase. A, developing ovule. B, enlargement of A, showing the funicular cortical cells with dense starch grains accumulated but four to five rows of smaller cells below the funicular distal end with only sparse starch grains. C, D, multicellular proembryo phase, showing the uppermost epidermal cells of funicular distal end with dense cytoplasm. E–G, globular embryo phase. E, developing ovule with an annular appendage around its base, appearing as two auriform outgrowths in section. F, enlargement of E, showing the much vacuolated epidermal cells and underlying cortical cells of the appendage, the former of which forming an arched surface. G, showing the funicular cortical cells with dense starch grains accumulated. H–J, heart-shaped embryo phase. H, developing ovule with the annular appendage around its base. I, enlargement of H, showing the thick-walled epidermal cells and the central vascular tissue which attaching funiculus to ovule. J, showing the epidermal cells with wall much thickened and funicular cortical cells with starch grains remarkably decreased. K–N, cotyledonary embryo phase. K, near mature ovule. L, enlargement of K, showing the extremely thick-walled epidermal cells and relatively constant underlying parenchymal cells. M, N, showing the annular appendage with its periphery attached funiculus hairs. O, a mature seed. P, enlargement of O, showing the hilar scar formed by detachment of the annular appendage from the seed. Scale bars: A, K, O, P = 100 μm; B, D, F, G, I, J, L, N = 20 μm; C, E, H, M = 50 μm. *Abbreviations*. EC, epidermal cell; F, funiculus; FC, funicular cortical cell; FDE, funicular distal end; FH, funicular hair; SG, starch grain; UC, underlying cell.

In fertilized ovules, during the octant proembryo phase (Fig 7), most of the cortical cells of the funiculus stained with PAS were dominated by shades of deep red due to the presence of densely accumulated starch grains. However, below the funicular distal end, which is at the junction of ovule body and funiculus, four to five rows of smaller cells only had sparse small starch grains (Fig 8A and 8B). Then in the following multicellular proembryo phase, the uppermost epidermal cells of the funicular distal end did not develop into hairs, but began to differentiate to form a distinct compact convex layer. These cells are rectangular in outline, about 8–10 μm long and 4–5 μm wide, characterized by their close arrangement and extremely dense cytoplasm, clearly distinguishable from other cells (Fig 8C and 8D). Subsequently, it is believed that both the epidermal cells and the underlying three to four rows of cortical cells reactivated meristematic activity and underwent periclinal divisions,which was verified by an increase in the number of cell layers (Fig 8E–8G). This meristem activity defined a zone of cells destined to take part in the formation of the annular appendage. In the globular embryo phase, cell proliferation disappeared but cell size began to increase as the appendage development progressed. The epidermal cells considerably increased their size to 17–20 μm long and 8–11 μm wide, extending downwards to form an arched surface (Fig 8E–8G). In parallel with the elongation, they became much vacuolated and thick-walled. Meanwhile, its underlying three to four rows of cortical cells also increased in size almost double and became more vacuolated (Fig 8E and 8F). This combination of increase in cell layers and cell size of both the epidermal and underlying cells together leads to producing a remarkable ring-shaped structure at the distal end of the funiculus, which is obviously thicker than the funiculus in diameter and showing two auriform outgrowths around the base of the developing ovule in longitudinal section (Fig 8E–8G). With the forming of the ring-shaped structure, the base of the almost mature seed (micropylar region) was covered by this ring and the seed was attached to the funiculus only by the ring’s central constricted zone (Fig 8E), which is about 1/3–1/4 the width of the ring and composed of vascular bundles surrounded by a thin zone of aerenchymatous tissue (Fig 8I).

From the heart-shaped embryo to cotyledonary embryo phase, the cell walls of the arched surface of the ring were increasingly thickened, which was verified by the red-stained cellulose, so that they protruded into the cell lumens and occupied most of them (Fig 8H–8N). In contrast, the underlying parenchymal cells remained more-or-less similar in size and shape (Fig 8I, 8J, 8L and 8N). During the heart-shaped embryo phase, another notable feature is that the accumulated starch grains in cortical cells of the funiculus significantly decreased compared to the earlier phase (Fig 8J), even almost disappearing in the cotyledonary embryo phase (Fig 8L).

**Fig 9:**
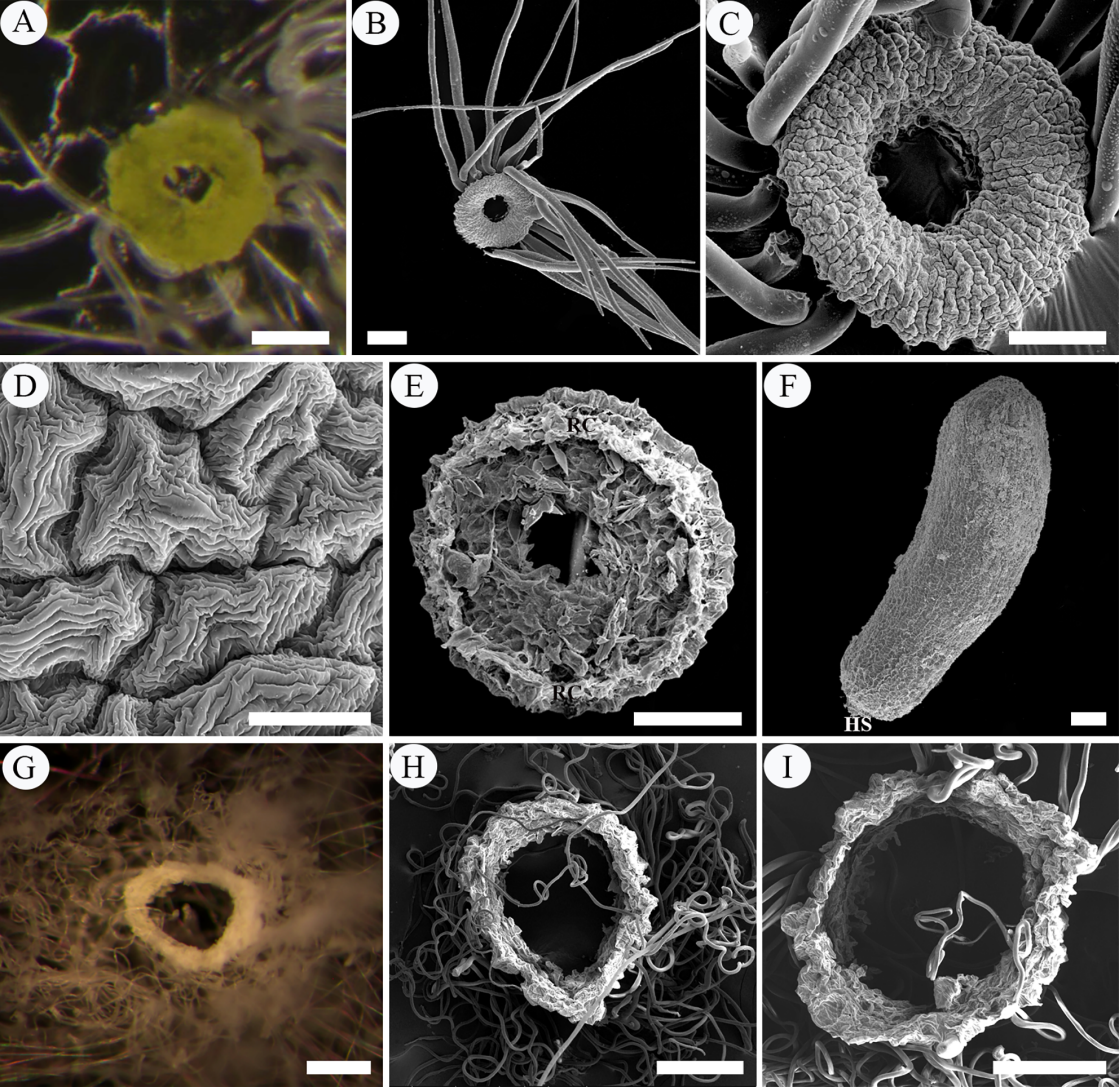
LM and SEM micrographs of mature annular appendage in *Salix matsudana*, *Populus* × *tomentosa* and *P*. *lasiocarpa*. A–F, *Salix matsudana*. A, LM, showing the outline of an annular appendage; B–F, SEM. B, annular appendage with some hairs attached. C, enlargement of B, showing the glabrous upper and side surface. D, enlargement of C, showing the surface cells with irregular shape, and extremely thickened and deeply wrinkled walls. E, underside, showing the rough surface with a raised circle. F, mature seed, showing the hilar scar formed by detachment of the annular appendage from the seed. G, H, *Populus* **×** *tomentosa*. G, LM, the outline of an annular appendage. H, SEM, annular appendage with many curly hairs attached. I, *Populus lasiocarpa*, SEM, annular appendage with many curly hairs attached. Scale bars: A–C, E–I = 100 μm; D = 10 μm. *Abbreviations*. HS, hilar scar; RC, raised circle.

When the seed ripens, the ring-shaped structure is separated from the top end of the funiculus, but is often still attached to the seed base. When the central vascular tissue of the ring is detached from the seed, a hilar scar is formed on the base of the seed (Figs 8O, 8P and 9F) and the ring is free (Figs 1B and 9A). Thus, the structural characteristics of the mature annular appendage can be observed, which is about 200–230 μm in diameter, and showing three different portions: a glabrous upper and side surrounding surface, a rough underside and a central circular hole (Fig 9B, 9C and 9E). The upper and side surface is arched and composed of a single layer of compact cells, which are characterized by their irregular shape, and extremely thickened and deeply wrinkled walls (Fig 9C and 9D). These cells arranged radially and there are altogether 54–56 radii on the whole surface, constituting a broad palisade like circle. Each radius is about 65 μm wide and consists of 10–12 cells (Fig 9C). By contrast, its underside is uneven with a raised thin circle near the edge of the ring, composed of many broken thin-walled cells resulting from the detachment with the funiculus (Fig 9E). The raised circle resulted from the remaining epidermis of the funiculus, so the periphery of the ring’s underside often bears many long hairs which produced by these epidermal cells, forming tufts of hairs under the ring (Fig 9B and 9C). The central hole is about one-third the diameter of the ring (Fig 9C and 9E), formed by the fracture of the vascular tissues between funiculus and mature seed.

### Appendage development in unfertilized ovules

**Fig 10:**
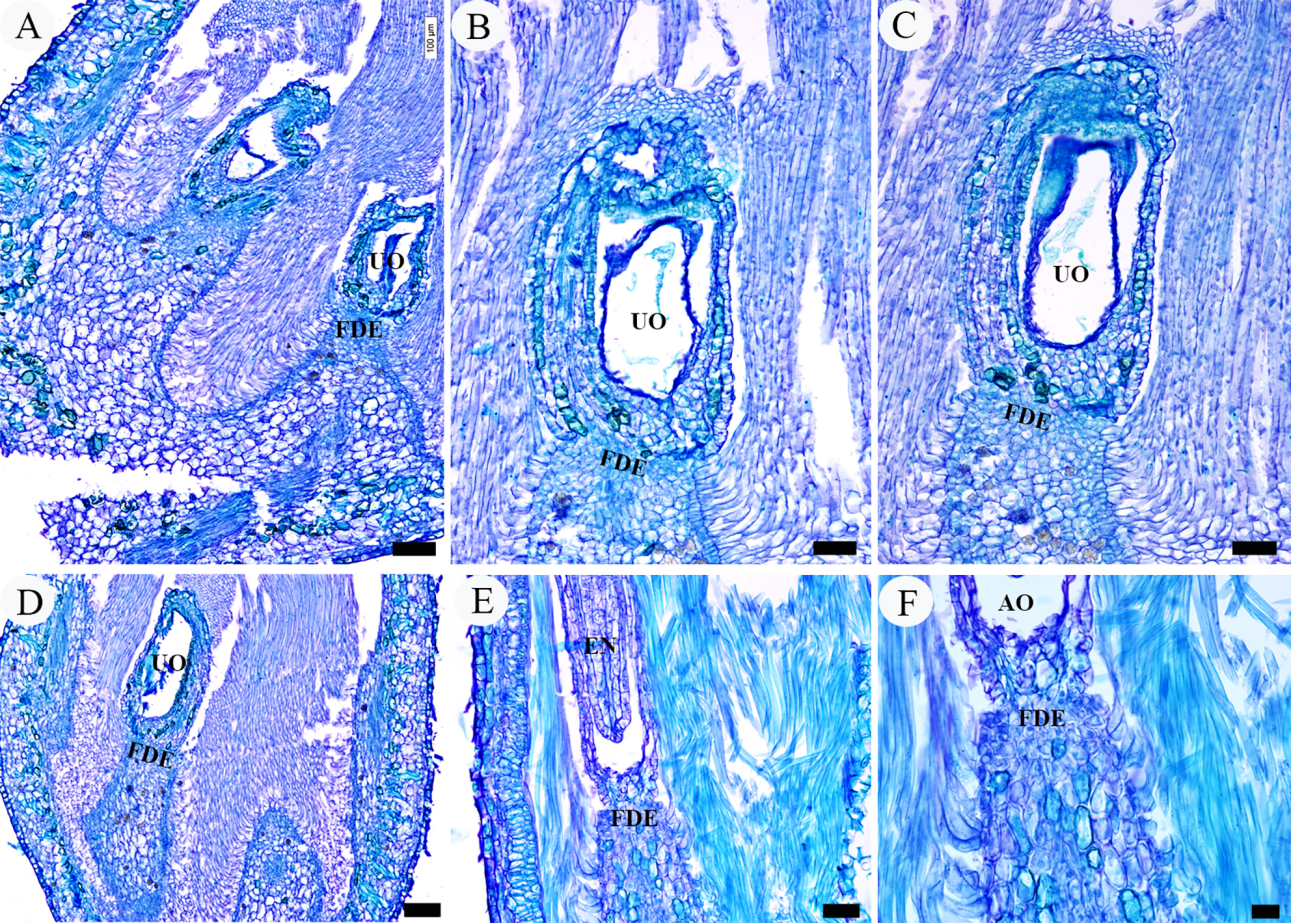
LM micrographs of appendages development in unfertilized ovules. A–D, unpollinated ovules, showing the hairs developed normally but cells of the funicular distal end remained unchanged and with no signs of the formation of annular appendage. E, F, a fertilized but aborted ovule, showing the same state as A–D. Scale bars: A–D = 100 μm; E = 50 μm; E = 20 μm. *Abbreviations*. AO, abortive ovule; EN, endosperm; FDE, funicular distal end; UO, unfertilized ovules.

After pollination, compared to those fertilized ovules, the bagged (unpollinated and unfertilized) ovules of *S*. *matsudana* exhibited only a slight increase in size, which are about one-half the length of the fertilized ovules, and then gradually degenerated (Fig 10A–10D). However, through our observations, their ovaries developed normally and the developmental features of the hairs seemed not to have clear differences between unpollinated ovules and fertilized ovules (Fig 10A–10D). These hairs also reached their full development, and no morphological and structural differences were found between the hairs in fertilized fruits and unfertilized fruits. As a result, although there is no developed seeds in unfertilized fruits, they were filled with the long hairs as well as those which had been fertilized.

On the other hand, obvious differences on origin and development of the annular appendage were observed. In the same period of the fertilized ovules, for those unpollinated ovules, the cells of the funicular distal end remained unchanged in size and shape, and there were no signs of the formation of the ring-shaped structure (Fig 10A–10D). Moreover, in one fertilized but aborted ovule in which there was normal endosperm but no developed embryo, also no signs of an annular appendage were found at the distal end of the funiculus (Fig 10E and 10F).

## Discussion

Seed appendages are the local outgrowths of the seed, often in the form of glandular swellings, ancillary pulpy envelopes or caps, wings and hair-tufts, which vary in morphology, origin, function, and chemical composition [9, 14, 21]. Although hairs are a relatively uncommon seed appendage, they are characteristics of some families such as Salicaceae (*s*. *str*.), Malvaceae (including Bombacaceae), Asclepiadaceae and Tamaricaceae [9, 14, 21]. In *Salix* and *Populus*, when the capsules mature and release the mature seeds, each seed is accompanied by numerous spreading whitish silky hairs, which exceed the length of the seed, and its base (radical end) is covered by a small annular appendage surrounded by tufts of hairs. In the present study, a set of morphological and anatomical observations provided a detailed overview of the development and structure of these two seed appendages in *S*. *matsudana* for the first time.

### Development of the intra-ovarian hairs

Based on our observations, the complete development of the hairs of *S*. *matsudana* consists of three stages: initiation, elongation, and maturation.

The initiation stage is characterized by the initiation of hair primordia. There have been different opinions about where the silky hairs surrounding the seeds of Salicaceae originated from. Most reports mentioned that the hairs in *Salix* or *Populus* originated from the placenta [8, 11, 12, 14, 16, 22]. On the other hand, a few studies described the hairs arising (also) from the funiculus [13, 17, 23]. The placental origin is partly due to the view that ovules of the two genera have no funicles [11, 14]. However, based on developmental observations, Fechner [13] described the ovules of *Populus tremuloides* (American aspen) as funicled, and Steyn et al. [17] concluded that the ovules of *Salix* possess distinct funiculus. In our study, through both LM and SEM, we also observed that each ovule of *S*. *matsudana* has a well-developed funiculus, and that the hair primordia initiated successively from the placenta epidermal cells and the epidermal cells of all sides of the funiculus. Therefore, we confirmed the hairs of *S*. *matsudana* have both a placental and funicular origin, and described them as placental hairs and funicular hairs separately in their development. In any case, they are unique and totally different from the more common seed hairs of many taxa, such as *Asclepias* [24], *Epilobium* [25], *Gossypium* [26], *Hibiscus* [27], *Hillia* [28] and *Tamarix* [29], in all of which the hairs are derived from the integument of the ovules.

As to the initiation time, the present study showed that the placental hairs of *S*. *matsudana* initiated very early in ovule development. In the megaspore mother cell phase of the megagametophyte or even earlier, the placenta epidermal cells around the funicular proximal end have begun to protrude above the placenta surface, forming the hair primordia. In contrast, the funicular hair primordia were initiated in the eight-nucleate phase, about ten days later than the placental hairs. In previous studies, only Nagaraj [12] reported that the seed hairs of *Populus tremuloides* were initiated at approximately the early eight-nucleate stage, which is almost simultaneous with the initiation of the funicular hairs of *S*. *matsudana*, but later than that of the placental hairs. We inferred that the initiation of the hairs in *Salix* is probably earlier than in *Populus*, but this needs further verification by a more extensive survey of the hair development in these two genera.

The elongation stage is characterized by a progressive length increase and vacuolization of the hair cells. Their elongation follows a sigmoid curve, with slow growth rate initially and a subsequent gradual rapid growth rate at later stages (Fig 7). The placental hairs elongated relatively slowly before the eight-nucleate phase and then began to grow faster and faster. The elongation of the funicular hairs started first at the bottom of the funiculus and gradually extended upwards. Before the multicellular proembryo phase, they elongated slowly but after that a very rapid development occurred. On the other hand, although the funicular hairs were initiated later than the placental hairs, the former displayed a more rapid elongation than the latter, with the result that both of them almost reached their final length at the same time.

In this study, it has been showed that each hair was derived from an individual epidermal cell. During its elongation, the nucleus which stained intensely, was observed continuously but without any evidence of cell division, so the unicellular state was maintained throughout the hair’s development. This property enables these hairs to be studied free of any influences from cell division, thus making them an ideal model for discovering mechanisms regulating cell elongation at the single-cell level.

As the hairs increased considerably in length, their cells became more and more vacuolated with the cytoplasm displaced to the periphery. However, in contrast to cells of the placenta and the cortical cells of the funiculus, cytoplasm in these hair cells were never found to contain starch grains throughout their development, indicating that the initiation and elongation of the hairs did not need the digestion of their own storage polysaccharides to supply nutrition and/or energy.

The maturation stage of the hairs is characterized by a change in shape and lignification of the cell walls, followed by their detachment from the placenta and funiculus. In the cotyledonary embryo phase, the shape of the hairs began to change from flattened to cylindrical. The red-purple staining with phloroglucinol indicated that there was abundant lignin in the hair cell walls, which supported the observations by Steyn et al. [17] in *S*. *mucronata*. When mature, the hairs consist of long, dead, air-filled cells with their walls lignified. When they detached and spread out from the fruits, the hairs were unassociated with the mature seeds but sometimes with bits of placenta or funiculus attached.

### Development of the annular appendage

Steyn et al. [17] were the first to briefly describe the initial and mature morphology of a small annular appendage, referred to as a “hilar aril”, in African willow *S*. *mucronata*. Compared to their observations, similar but more detailed observations based on a complete ontogeny of this appendage were obtained in current study. Based on our observations, the complete development of the annular appendage in *S*. *matsudana* can be divided into three developmental stages: initiation, thickening, and maturation.

The initiation stage is characterized by the development of the appendage primordium. Steyn et al. [17] noted that in *S*. *mucronata*, during the elongation of the embryo sac, a plate-like intercalary meristem developed in the distal part of the funiculus, but did not mention the exact initiation period and the cell characteristics of the meristem. The current study showed that during the octant proembryo phase in fertilized ovules, four to five rows of smaller cells at the distal end of the funiculus, which are characterized by having less starch grains, become meristematic. Then in the following multicellular proembryo phase, the uppermost epidermal cells at this end began to increase their cytoplasm, forming a single convex layer below the ovule body. Subsequently, both the single layer of epidermal cells and underlying three to four rows of cells underwent periclinal divisions, forming the appendage primordium.

The thickening stage is characterized by the appendage cells undergoing a considerable size increase and vacuolation. During the globular embryo phase, both the epidermal and underlying cells did not increase in number but approximately doubled in size, accompanied by their vacuolation with the large central vacuole occupying most of the cell lumen. Another feature at this stage is an increasing thickening of the epidermal cell walls. Interestingly, from the globular embryo to heart-shaped embryo phase, this cell wall thickening and the significant decrease of starch grains in the funicular cortical cells took place simultaneously, suggesting that the storage polysaccharides may probably be correlated with the synthesis of cellulose in the cell wall.

The maturation stage is characterized by a continuous increase in thickness of the upper surface and detachment of the underside from the funiculus. In the cotyledonary embryo phase, the walls of the epidermal cells have thickened so much that their large central vacuoles were extremely squeezed by the thicken walls. As a result, they became more compactly arranged and changed their shape from rectangular to irregular, forming a thick and solid upper surface of the annular appendage. At the same time, the underlying cells began to detach from the funiculus, forming a rough lower surface. Steyn et al. [17] noted that in mature seed of *S*. *mucronata*, the annular structure had a glabrous upper surface and a rough lower surface, and numerous long hairs attached to its perimeter, forming the plume or coma on the seed. These observations agree with the situation in *S*. *matsudana*, and the hairs, which actually originating from funiculus epidermal cells, were also observed on the periphery of the appendage underside in *S*. *matsudana*. However, because of a lack of information on the developmental and structural details of this annular appendage in *S*. *mucronata*, we can not make further comparison between the two species.

### Developmental correlation between appendages and ovules

The results of the present study showed that the placental and funicular hairs of *S*. *matsudana* were both initiated before ovule fertilization, and that unpollinated and aborted ovules developed abnormally but produced normal hairs, indicating that the initiation, elongation and maturation of the hairs were independent of ovule development. This observation is in agreement with previous reports of the hairs in several species of *Salix* and *Populus* [11, 12, 17]. Therefore, it means that the formation and development of the hairs are independent from both fertile and aborted ovules in these two genera, and in all instances, the hairs will be released from the mature fruits. Furthermore, these hairs were derived from the epidermal cells of the placenta and funiculus and were not present on the ovule body, thus in the mature seeds the hairs are associated with but not physically attached to, the seeds.

On the other hand, for the annular appendage, our observations showed that it was initiated soon after ovule fertilization, and if fertilization did not take place the cells at distal end of the funiculus remained practically unchanged. Also, there were no signs of the formation of a ring-shaped structure in unpollinated or aborted ovules until they degraded, indicating that fertilization and ovule development are a necessarily prerequisite for the annular appendage to occur. Here, we inferred that the normal embryo development may reactivate the meristem capacity of the annular appendage primordium. However, how the fertilization and subsequent embryo development acted on the annular appendage cells remains unknown and needs further study.

### Evolutionary significance of the seed appendages

Steyn *et al*. [17] provided an observation on the structure of seed and seed appendages in *S*. *mucronata*, as well as their possible functional significance during seed dispersal. Compared with this African willow species, similar observations on the morphology and structure of seed appendages in Chinese willow *S*. *matsudana* were obtained. The mature seeds of both species were characterized by numerous long hairs which loosely accompanying the seeds, and a tiny annular structure with tufted hairs surrounding the base of the seed, resulting in a unique comose seeds. Clearly this kind of comose seeds are one of the diagnostic features of the genus *Salix*. Moreover, we compared the mature seeds of two species of *Populus*, namely *P*. × *tomentosa* and *P*. *lasiocarpa*, finding that besides the apparent scattered hairs, each seed of both poplar species also possessed a small annular appendage with tufts of hairs surrounding its base. Therefore, it is confirmed that *Salix* and *Populus* shared common characteristics regarding seed appendages. For a long time, it was uncertain whether *Salix* and *Populus* are a natural, monophyletic group [30]. Recent phylogenetic studies based on combined molecular data suggested that the two genera form a truly monophyletic group [30]. The present study provides structural evidence from seed appendages, supporting that *Salix* and *Populus* are closely related sister genera and share a most recent common ancestor. In general morphology, the annular appendage in *Populus* is similar to that of *Salix*, but appear more simple with a much narrower and reduced upper surface and larger central hole, and the hairs attached around the underside are usually curly (Figs 1C and 9G–9I). The detailed structural difference and evolutionary relationship of this appendage between the two genera require further investigation.

Evolutionary, the Salicaceae has a rich fossil record [31] and the earliest confirmation of the group is in the early Middle Eocene (ca. 48 million years ago), when vegetative and/or reproductive organs confirm the presence of the extant genus *Populus* and extinct genus *Pseudosalix* from North America [32, 33]. Among these fossils, some well-preserved leaves and fruits was described as a fossil species *Populus tidwellii* Manchester et al., although its placement remained suspect because it possesses *Populus*-like infructescences but *Salix*-like leaves [33]. Most importantly, some dispersed hairy seeds attributed to *P*. *tidwellii* were also found. These fossil seeds were elliptical, small, comose and dispersed with longer cottony placenta/funiculus, similar to the modern comose seeds of *Populus* and *Salix*. This indicated that by the early Middle Eocene, the feature of Salicaceae seeds surrounded with long hairs was already present and is an ancestral character. On the other hand, from the description and figures of these fossil seeds provided, it seemed that the numerous long hairs did not detach from its funiculus but dispersed while still attached to the funiculus. Also, there was no annular appendage reported from the fossils. Therefore, we inferred that the detachment of hairs from the funiculus and the occurrence of an annular appendage with tufts of hairs probably are the more derived states for seed dispersal in *Populus* and *Salix*.

## References

[1] Chase MW, Zmarzty S, Lledó MD, Wurdack KJ, Swensen SM, Fay MF. When in doubt, put it in Flacourtiaceae: a molecular phylogenetic analysis based on plastid rbcL DNA sequences. Kew Bull. 2002;57: 141–181.

[2] Soltis DE, Soltis PS, Endress PK, Chase MW. Phylogeny and evolution of angiosperms Sunderland: Sinauer Associates; 2005.

[3] Argus GW. Infrageneric classification of New World *Salix* L. (Salicaceae). Syst Bot Monogr. 1997;52: 1–121.

[4] Fang ZF, Zhao SD, Skvortsov AK. Salicaceae In: Wu Z, Raven PH, editors. Flora of China vol. 4 St. Louis, MO: Missouri Botanical Garden Press;1999.

[5] Jordaan M. *FSA* contributions 18: Salicaceae s. str. Bothalia. 2005;35(1): 7–20.

[6] Argus GW. Salicaceae Willow Family: Part Two: *Salix* L. Willow. Journal of the Arizona-Nevada Academy of Science. 1995;29(1): 39–62.

[7] Keoleian GA, Volk TA. Renewable energy from willow biomass crops: life cycle energy, environmental and economic performance. Crit Rev Plant Sci. 2005;24: 385–406.

[8] Van der Pijl L, Principles of dispersal in higher plants. Third Edition Berlin: Springer-Verlag; 1982.

[9] Werker E. Seed Anatomy. Berlin and Stuttgart: Gebrüder Borntraeger; 1997.

[10] Hu YQ, Ferguson DK, Bera S, Li CS. Seed hairs of poplar trees as natural airborne pollen trap for allergenic pollen grains. Grana. 2008;47: 241–245.

[11] Takeda H. On the coma or hairy tuft on the seed of willows. Bot Mag Tokyo. 1936;50: 283–289.

[12] Nagaraj M. Floral morphology of *Populus deltoides* and *P*. *tremuloides*. Bot Gaz. 1952;114(2): 222–243.

[13] Fechner GH. Development of the pistillate flower of *Populus tremuloides* following controlled. Can J Bot. 1972;50: 2503–2509.

[14] Corner EJH. The seeds of dicotyledons. Cambridge: Cambridge University Press; 1976.

[15] Argus GW. The Genus *Salix* (Salicaceae) in the Southeastern United States. Sys Bot Monog. 1986;9: 1–170.

[16] Boes TK, Strauss SH. Floral phenology and morphology of black cottonwood, *Populus trichocarpa* (Salicaceae). Amer J Bot. 1994;81(5): 562–567.

[17] Steyn EMA, Smith GF, van Wyk AE. Functional and taxonomic significance of seen structure in *Salix mucronata* (Salicaceae). Bothalia. 2004;34(1): 53–59.

[18] Takhtajan A. Flowering Plants. Second Edition Dordrecht: Springer Netherlands; 2009.

[19] O’Brien TP, McCully ME. The study of plant structure principles and selected methods Melbourne: Termarcarphi Publishers; 1981.

[20] Yeung EC. Histological and histochemical staining procedures In: Vasil IK, ed. Cell culture and somatic cell genetics of plants. Orlando, FL: Academic Press; 1984.

[21] Kapil RN, Bor J, Bouman F. Seed appendages in angiosperms. Nische Jahrbücher für Systematik. 1980;101: 555–573.

[22] Johri BM, Ambegaokar KB, Srivastava PS. Comparative embryology of angiosperms. vol.1 London: Springer-Verlag; 1992.

[23] Jacobs M. Salicaceae. Flora Malesiana, Series 1, Spermatophyta: flowering plants. Vol. 5. Djakarta: Noordhoff-Kolff NV; 1955.

[24] Pearson NL. Observations on seed and seed hair growth in *Asclepias syriaca* L. Amer J Bot. 1948;35(1): 27–36.

[25] Coşkunçelebi K, Makbul S, Okur S. Seed morphology of *Epilobium* and *Chamaenerion* (Onagraceae) in Turkey. Phytotaxa. 2017;331(2): 169–184.

[26] Flint EA. The structure and development of the cotton fibre. Biol Rev. 1950;25(4): 414–434.

[27] Kumar P, Singh D. Development and structure of seed-coat in *Hibiscus* L. Phytomorphology. 1990;40: 179–188.

[28] Puff C, Buchner R. Development and structure of the comose seeds of *Hillia* (Rubiaceae). Plant Syst Evol. 1998;210: 147–157.

[29] Johri BM, Kak D. The embryology of *Tamarix* Linn. Phytomorphology. 1954;4: 230–247.

[30] Liu X, Wang Z, Wang D, Zhang J. Phylogeny of *Populus*–*Salix* (Salicaceae) and their relative genera using molecular datasets. Biochem Syst Ecol. 2016;68: 210–215.

[31] Collinson ME. The early fossil history of Salicaceae–a brief review. P Roy Soc Edinb B. 1992;98: 155–67.

[32] Boucher LD, Manchester SR, Judd WS. An extinct genus of Salicaceae based on twigs with attached flowers fruits, and foliage from the Eocene Green River Formation of Utah and Colorado, USA. Amer J Bot. 2003;90: 1389–1399. 21659238 10.3732/ajb.90.9.1389

[33] Manchester SR, Judd WS, Handley B. Foliage and fruits of early poplars (Salicaceae: *Populus*) from the Eocene of Utah, Colorado, and Wyoming. Int J Plant Sci. 2006;167: 897–908.

